# Supplementary material for: Chromothripsis during telomere crisis is independent of NHEJ, and consistent with a replicative origin
Source: Genome Res. 2019 May;29(5):737–49. doi: 10.1101/gr.240705.118 (PMC6499312; doi:10.1101/gr.240705.118)
Supplement: Supplemental Material [file supp_gr.240705.118_Supplemental_file_1.zip › contigs/annotated_contigs/DB82/contig.3.DB82_length_411_mean_cov_9.3503649635.docx]

**DB82_length_411_mean_cov_9.3503649635**

```
GTGGCGGGCGCCTGTAATCCTAGCTACTTGGGAGGCTGAGGCAGGAGAATCACTGGAAACCGGGAGGCAGAGGTTGCAGTGACCCGAGA
 >chr10:70691182-70691350 - E=8e-82 p=0e+00
CTGTGCCACTGCACTCCAGCCTGGGCAACAGAGCAAAATTCCATCTCAAAAAAAAATTGTGTGTGTGTGTGTGTGTGT|GTGTGTGTGT

GTGTGTGTGTGTG|TATATATATATATATATTTTTTTTTTAATGATCCGTCACCCACCCACCAGCCTGGGTGACAGAGCAAGACCCTGT
              >chr10:70691067-70691165 - E=1e-42
TTCTGAAAAAGGAAATAAAGA|G|TTTTGGCTTAAAAGGGCAGGATATCTTGAAGGGGCTTACAGATCGTAGGTACATTTAAAGATTTT
                      >chr10:70634131-70634256 - E=1e-63
CTATTTTAATTGGTTAAGGAAGAGAAGCTTTGTTTAAAAGTCTGGGGTCAGTAACTGGC
```
